# Risk Factors for Reduced Salivary Flow Rate in a Japanese Population: The Hisayama Study

**DOI:** 10.1155/2015/381821

**Published:** 2015-02-03

**Authors:** Kenji Takeuchi, Michiko Furuta, Toru Takeshita, Yukie Shibata, Yoshihiro Shimazaki, Sumio Akifusa, Toshiharu Ninomiya, Yutaka Kiyohara, Yoshihisa Yamashita

**Affiliations:** ^1^Section of Preventive and Public Health Dentistry, Division of Oral Health, Growth and Development, Faculty of Dental Science, Kyushu University, Fukuoka 812-8582, Japan; ^2^Department of Preventive Dentistry and Dental Public Health, School of Dentistry, Aichi Gakuin University, Aichi 464-8650, Japan; ^3^Department of Health Management, School of Oral Health Science, Kyushu Dental College, Kitakyushu 803-8580, Japan; ^4^Division of Research Management, Center for Cohort Studies, Graduate School of Medical Sciences, Kyushu University, Fukuoka 812-8582, Japan; ^5^Department of Environmental Medicine, Graduate School of Medical Sciences, Kyushu University, Fukuoka 812-8582, Japan

## Abstract

The purpose of this study was to determine distinct risk factors causing reduced salivary flow rate in a community-dwelling population using a prospective cohort study design. This was a 5-year follow-up survey of 1,377 community-dwelling Japanese individuals aged ≥40 years. The salivary flow rate was evaluated at baseline and follow-up by collecting stimulated saliva. Data on demographic characteristics, use of medication, and general and oral health status were obtained at baseline. The relationship between reduced salivary flow rate during the follow-up period and its predictors was evaluated after adjustment for confounding factors. In a multivariate logistic regression model, higher age and plaque score and lower serum albumin levels were significantly associated with greater odds of an obvious reduction in salivary flow rate (age per decade, odds ratio [OR] = 1.25, 95% confidence interval [CI] = 1.03–1.51; serum albumin levels <4 g/dL, OR = 1.60, 95% CI = 1.04–2.46; plaque score ≥1, OR = 1.53, 95% CI = 1.04–2.24). In a multivariate linear regression model, age and plaque score remained independently associated with the increased rate of reduced salivary flow. These results suggest that aging and plaque score are important predictors of reduced salivary flow rate in Japanese adults.

## 1. Introduction

Reduced salivary flow rate impedes the mastication and swallowing of food, neutralization of acids, and immunological protection against potential pathogens [1, 2]. Hyposalivation resulting from reduced salivary flow rate leads to oral diseases such as dental caries and mucosal lesions [3]. In addition, subsequent hyposalivation-associated problems such as dysphagia also result in reduced quality of life [4, 5]. Thus, reduction in salivary flow may be a fundamental factor of the above-mentioned disorders. In older individuals, complaints of oral dryness resulting from reduced saliva are common [5, 6]. According to a recent report, the prevalence of subjective dry mouth and reduced stimulated salivary flow rate was significantly higher in older individuals than in younger individuals [7] in Japan, which has a very high proportion of older individuals. However, there is no effective or definitive therapy to reverse oral dryness. Therefore, early detection of risk factors for reduced salivary flow rate is important for maintaining both oral and general health in the currently aging society.

Many factors have been identified as possible causes of reduced salivary flow rate, including medications and systemic diseases [8–11]. In addition, it has been reported that aging, nutritional or general health status, number of remaining teeth, periodontal condition, and bite force are correlated to salivary flow rate [12–17]. However, to our knowledge, no study has revealed distinct risk factors for reduced salivary flow rate with a prospective study design. Therefore, we analysed the relationship between an obvious reduction in salivary flow rate over 5 years of follow-up and its predictors to identify distinct risk factors causing reduced salivary flow rate.

## 2. Material and Methods

### 2.1. Study Population

The present study is based on data from the Hisayama Study, which is a population-based prospective cohort study of cardiovascular disease and its risk factors established in 1961 in the town of Hisayama, a suburb of the Fukuoka metropolitan area in Kyushu, Japan [18]. Based on national census data, the age and occupational distributions in Hisayama have been almost identical to those across Japan since the 1960s [19]. Briefly, as a part of the study, two cross-sectional surveys were conducted on Hisayama residents in a similar manner in 2007 and 2012. In 2007, 2,930 residents aged 40 years and older (67.7% of the total number of residents in this age group) consented to participate in dental and medical examinations and underwent a comprehensive health assessment for the present study. After a 5-year follow-up (observation period, June 2007 to October 2012), 2,176 residents remained in the cohort (response rate, 74.3%). The following residents who were not able to provide sufficient data for proper assessment of the rate of reduced salivary flow were excluded: 662 residents with missing responses regarding information on salivary flow rate or missing an oral examination; 52 residents who had previously used antidepressants, antihistamines, or muscle relaxant agents; and 85 residents who were previously diagnosed with Sjögren syndrome or chronically low salivary flow rate (<1.0 mL/min stimulated whole saliva) [20, 21] at baseline. Thus, the remaining 1,377 participants (595 men and 782 women) formed the final population of this 5-year cohort study. The study was approved by the Kyushu University Institutional Review Board for Clinical Research. Written informed consent was obtained from all participants.

### 2.2. Evaluation of Salivary Flow Rate

Stimulated saliva was collected to accurately diagnose a reduction in salivary flow instead of resting saliva, which has been reported to be more sensitive to taking medications [22]. For sampling, we used a simple design method that can be measured in both clinical setting and group medical examination easily in a short time. Sampling was always conducted in the period from June to October between 8:00 am and 10:30 am during morning fasting in 2007 (baseline) and 2012 (follow-up). Participants were asked to sit in a chair and chew sugar-free xylitol gum for 2 min so that stimulated saliva could be collected. Participants expectorated the accumulated saliva into a sterile tube. After 2 min, the volume of collected saliva was measured, and the salivary flow rate was expressed in mL/min.

### 2.3. Outcome Variable

The outcome measure for the analysis was the rate of reduction in salivary flow during the 5-year follow-up period, which was the amount of reduction in the salivary flow rate in the follow-up period as a fraction of the initial salivary flow rate. In a previous report, the lowest 10th percentile for all recorded stimulated salivary flow rates in all age and sex cohorts was regarded as low or indicative of stimulated salivary flow rates outside the generally accepted range of values for healthy individuals [23]. Accordingly, we used the top 10th percentile of the distribution of the rate of reduction in salivary flow (corresponding to a ≥35% reduction in salivary flow rate) as indicative of an obvious reduction in salivary flow rate. Thus, in the logistic regression model, we used obvious reduction in salivary flow rate as a dichotomized variable (1 = ≥35% reduction in salivary flow rate, 0 = <35% reduction in salivary flow rate). In addition, in the linear regression model, we used rate of reduction in salivary flow as a continuous variable.

### 2.4. Predictor Variables

We included established or supposed factors associated with reduced salivary flow rate. Information on demographic characteristics, medical history of stroke (yes or no), use of antihypertensive agents (yes or no), smoking habits (current, ever, or nonsmoker), alcohol intake (current, ever, or nondrinker), and tooth brushing frequency (0, 1, 2, or ≥3 times a day) was obtained at baseline using a self-administered questionnaire. We dichotomized smoking habits and alcohol intake into the two categories of current and noncurrent. We also dichotomized tooth brushing frequency into “once a day” and “twice or more a day.” Body height and weight were measured, and body mass index (BMI) was calculated and dichotomised as <25 or ≥25.

A blood sample was collected from the antecubital vein in the morning after an overnight fast and analysed for serum albumin and plasma glucose levels. Serum albumin levels <4 g/dL were defined as low [24]. Using an oral glucose tolerance test, diabetes mellitus was defined as plasma glucose levels of 200 mg/dL or higher 2 h after ingestion of the glucose load [25].

Oral examination was performed by calibrated dentists, following the method of the Third National Health and Nutrition Examination Survey [26]. Dental prosthetic status was categorised as ≥20 remaining natural teeth with or without dentures, or <20 remaining natural teeth with or without dentures, according to a previous report [27]. Measurement of the dental plaque score by the Silness and Löe plaque index [28] was based on evaluations of both soft debris and mineralised deposits on 6 selected teeth (positions 16, 12, 24, 36, 32, and 44.). Each of the 4 surfaces of the teeth (buccal, lingual, mesial, and distal) was given a score from 0 to 3. Scores from the 4 areas of the tooth were summed and divided by 4 to give the plaque index for the tooth. The plaque score for the individual, obtained by summing the indices for all 6 teeth and dividing by 6, was dichotomised as <1 or ≥1. Periodontal status was assessed using probing pocket depths (PPD) and clinical attachment levels (CAL) on the mesiobuccal and midbuccal sites for all teeth except the third molars. We defined periodontitis as the presence of ≥1 site with both PPD and CAL of ≥4 mm, based on a previous report [29].

### 2.5. Statistical Analysis

Descriptive analyses were used to characterize the baseline characteristics of participants. To examine the relationship between obvious reduction in salivary flow rate during the follow-up period and its predictors, the odds ratio (OR) and 95% confidence interval (CI) were calculated using binomial logistic regression. In logistic regression models, salivary flow rate and age were included as continuous variables. In the univariate model (model 1), we calculated the crude OR and 95% CI for obvious reduction in salivary flow rate based on the predictor variables. In the multivariate model (model 2), we included sex and all other variables with a significance level of *P* < 0.05 in the univariate model. Additionally, we evaluated whether there was a similar relationship between obvious reduction in salivary flow rate and its predictors even when using stepwise multivariate linear regression analysis. Sex and all other variables that showed statistical significance in the univariate analysis of the rate of reduction in salivary flow (*P* < 0.05) were entered into the model. In addition, we calculated the population attributable fraction (PAF) of the obvious reduction in salivary flow rate attributable to each predictor variable. The PAF is generally defined as the reduction in the risk factor that would be achieved if the population had been entirely unexposed, compared with its current exposure pattern [30, 31]. All analyses were performed using SPSS statistical software version 21 (SPSS Inc., Chicago, IL, USA) with a significance level of 5%.

## 3. Results

Compared with the included subjects (*n* = 1,377), those excluded (*n* = 799; data not shown) were significantly older. There were no significant differences in sex ratio between the included and excluded subjects. The baseline characteristics of the study participants (*n* = 1,377; average age = 59.3 ± 9.9 years for men and 58.0 ± 9.9 years for women) are shown in Table 1. Of the participants, 20.5% belonged to the 40–49-year-old age group, 35.0% to the 50–59-year-old age group, 29.2% to the 60–69-year-old age group, and 15.3% to the >70-year-old age group. For salivary flow rate, 19.9% of the participants belonged to the 1.0–2.0 mL/min group, 29.4% to the 2.0–3.0 mL/min group, 27.7% to the 3.0–4.0 mL/min group, and 23.0% to the ≥4.0 mL/min group. Participants in the younger age groups and men tended to belong to the higher salivary flow rate groups.

The results of the logistic regression analysis for obviously reduced salivary flow rate and its predictors are shown in Table 2. In the univariate model (model 1), obviously reduced salivary flow rate was significantly associated with age, medical history of stroke, serum albumin levels, dental prosthetic status, plaque score, and periodontal status. In the multivariate model (model 2), higher age and plaque score and lower serum albumin levels remained significantly associated with greater odds of obvious reduction in salivary flow rate after adjustment for other confounding variables (age per decade, OR = 1.25, 95% CI = 1.03–1.51; serum albumin levels <4 g/dL, OR = 1.60, 95% CI = 1.04–2.46; plaque score ≥1, OR = 1.53, 95% CI = 1.04–2.24). Even in the multivariate linear regression model (Table 3), age and plaque score remained independently and positively associated with rate of reduction in salivary flow (age per decade, *β* = 0.058, *P* < 0.001; plaque score, *β* = 0.065, *P* = 0.012). In addition, salivary flow rate and sex were independently and positively associated with rate of reduction in salivary flow (salivary flow rate, *β* = 0.116, *P* < 0.001; sex, *β* = 0.116, *P* < 0.001).

The percentage of obvious reduction in salivary flow rate in each predictor and the PAFs is shown in Table 2. Overall, 150 participants (10.9%) showed an obvious reduction in salivary flow rate during the follow-up period. The PAFs for serum albumin levels and plaque score that were significantly associated with obvious reduction in salivary flow rate were 8.0% and 15.6%.

## 4. Discussion

This population-based 5-year cohort study demonstrated that higher age and plaque score and lower serum albumin levels were significantly associated with increased risk of obviously reduced salivary flow rate during the follow-up period. These associations remained significant even after adjustment for other confounding factors. In addition, our results indicate that the rate of reduction in salivary flow during the follow-up period increased as age and plaque score increased. To our knowledge, this is the first prospective study to comprehensively evaluate the risk factors for obviously reduced salivary flow rate in a relatively large sample of community living adults.

Our findings agree with those of previous studies indicating that reduced salivary flow rate is independent of age-related changes and rather reflects secondary systemic changes related to medications and diseases [32, 33]. However, in our results, higher age was associated with a higher risk of reduced salivary flow rate, adjusted for intake of medications and general condition, possibly because we could not fully control for potential confounding factors, such as newly taken medications and diagnosed systemic disorders. In particular, we could not rule out the possibility that older participants may have started taking new medications or may have been newly diagnosed with a systemic disorder since our study started in 2007. The increase in medication use that occurs with increasing age has been reported to be negatively associated with salivary flow rate [34]. Similarly, the increased prevalence of systematic disorders with increasing age may also have been a confounding factor. In our analysis, medical history of stroke, serum albumin levels, dental prosthetic status, plaque score, and periodontal status were related to both age and reduction in salivary flow rate. These results are generally consistent with prior studies indicating that as age increases, risk factors linked to reduced salivary flow rate increase [35, 36]. However, the influence of potential unadjusted confounding factors such as previous chemo- or radiotherapy exposure as part of head and neck cancer treatment should be evaluated further.

In the present study, the risk of reduced salivary flow rate increased with higher plaque scores, but there was no clear link between salivary flow rate and oral hygiene. One possible mechanism for this association is that a plaque accumulation includes a high concentration of bacterial lipopolysaccharides (LPS), which increase prostaglandins in salivary glands, blocking salivary secretion. This sequence of events leading from LPS exposure to inhibition of salivary secretion was confirmed by others in a previous report using an animal model [37]. Thus, a high plaque score as representative of a poor oral hygiene may lead to decreased secretion of saliva.

We confirmed that lower serum albumin levels as potential risk factors causing reduced salivary flow rate. The positive correlation of serum albumin levels to salivary flow rate is consistent with a previous study [38]. This relationship may be explained by the following mechanism. Serum albumin levels are generally considered as a practical marker of general health or of nutritional status [24]. As described above, increased number of medications used in association with poor general health status may lead to a reduced salivary flow rate [8]. However, we cannot confirm this mechanism even if we adjust for baseline medication use because we could not detect newly increased numbers of medications taken in our cohort model.

The results of our study have public health implications. We are interested in estimating the PAFs associated with risk factors for obvious reduction in salivary flow rate. The plaque score had the highest PAF, which implies that in 15.6% of cases in the population, the onset of obvious reduction in salivary flow rate may be attributed to a plaque score ≥1. Therefore, promoting regular dental visits or consistent home dental care to maintain the plaque score at <1 is a public health intervention that may aid in preventing reduction in the salivary flow rate.

A strength of our study is the prospective cohort design, which established the presence of poor oral hygiene and low serum albumin levels prior to the onset of obvious reduction in salivary flow rate. The relatively large sample from a representative general population-based group and the capacity to adjust for several possible confounders can be considered additional strengths because they allowed us to generalize our results. We recognize several limitations of our study as well. First, our follow-up rate was just under 80%; hence, our findings may not be well representative of the area in which the surveys were conducted. However, the follow-up rate in our cohort can be regarded as relatively high based on information from the Oxford Centre for Evidence-Based Medicine [39]. However, considering that one of the objectives of this study was to contribute to the prevention of reduced salivary flow rate, our findings are not greatly affected by lack of representativeness because we made valid comparisons within a relatively large cohort of community living adults [40]. Second, the excluded subjects were older than the included subjects. Therefore, our findings may not be directly applicable to older populations. Third, as already mentioned, the lack of information regarding potential confounding factors related to reduced salivary flow rate, such as previous chemo- or radiotherapy exposure as part of head and neck cancer treatment, may have reduced the accuracy of our findings to some extent. However, if these unmeasured factors affect the obvious reduction in salivary flow rate and its predictors, a baseline adjustment would reduce the confounding effects [41]. Therefore, in the multivariate logistic and linear regression models, we included the baseline salivary flow rate as a baseline adjustment to control for undetermined confounding variables. Fourth, saliva collection was performed 5 years apart with only a single measure of salivary flow rate. Therefore, it is not possible to conclusively determine whether the reduction in salivary flow rate is stable over time or only represents normal variation. Additionally, the 5-year period can be regarded as rather short for observation in changes in salivary flow rate. Future studies should evaluate decadal changes in this population. Fifth, we did not obtain monitoring data for the plaque score and serum albumin levels at time points other than the baseline. Therefore, although our analysis included prospective cohort data, we cannot account for potential changes in patterns of plaque scores and serum albumin levels during the 5-year follow-up period. Additionally, it is possible that some individuals were lost from baseline to follow-up because of reduced propensity for healthier behaviour. Thus, our results may not have captured individuals who developed poor oral hygiene. Therefore, these findings should be interpreted with care.

## 5. Conclusions

The present study indicates that aging and plaque score are important predictors of reduced salivary flow rate in Japanese adults. These findings highlight that maintaining good oral hygiene may have not only a conventional clinical value but also long-term health implications for the prevention of reduced salivary flow rate with increasing age.

## Figures and Tables

**Table 1:** Baseline characteristics of participants (*n* = 1,377).

	*n*	%	Age (mean ± SD)	Men (%)
Total	1,377	100.0	58.6 ± 9.9	43.2
Salivary flow rate (mL/min)				
1.0–2.0	274	19.9	59.1 ± 10.3	27.4
2.0–3.0	405	29.4	59.5 ± 9.6	32.1
3.0–4.0	381	27.7	58.6 ± 9.8	47.0
≧4.0	317	23.0	56.9 ± 10.1	66.6
Age				
40–49	282	20.5	44.9 ± 2.9	37.9
50–59	482	35.0	55.0 ± 2.9	43.6
60–69	402	29.2	64.3 ± 2.8	42.3
≧70	211	15.3	74.1 ± 3.7	51.2
Medical history of stroke				
Yes	32	2.3	65.9 ± 7.1	56.3
No	1,345	97.7	58.4 ± 9.9	42.9
Use of antihypertensive agent				
Yes	339	24.6	64.6 ± 8.8	51.3
No	1,038	75.4	56.6 ± 9.5	40.6
Smoking habits				
Current smoker	251	18.2	54.9 ± 8.6	79.3
Noncurrent smoker	1,126	81.8	59.4 ± 10.0	35.2
Alcohol intake				
Current drinker	753	54.7	57.7 ± 9.8	59.9
Noncurrent drinker	624	45.3	59.7 ± 10.0	23.1
Tooth brushing frequency				
Once a day	416	30.2	59.2 ± 10.4	64.7
Twice or more a day	957	69.5	58.3 ± 9.8	33.8
Missing	4	0.3	59.5 ± 4.2	75.0
BMI				
≧25	373	27.1	58.5 ± 9.6	48.3
<25	1,004	72.9	58.6 ± 10.1	41.3
Serum albumin (g/dL)				
Normal (≧4)	1,168	84.8	58.2 ± 9.8	43.2
Low (<4)	209	15.2	60.8 ± 10.3	43.1
2 h postload plasma glucose levels (mg/dL)				
Diabetes (≧200)	141	10.2	63.2 ± 9.7	63.1
Nondiabetes (<200)	1,213	88.1	57.9 ± 9.8	41.1
Missing	23	1.7	64.8 ± 8.8	34.8
Dental prosthetic status				
≧20 remaining natural teeth with dentures	92	6.7	64.8 ± 8.4	50.0
≧20 remaining natural teeth without dentures	1,091	79.2	56.7 ± 9.4	42.6
<20 remaining natural teeth with dentures	150	10.9	67.3 ± 8.5	41.3
<20 remaining natural teeth without dentures	44	3.2	61.8 ± 8.1	50.0
Plaque score				
≧1	365	26.5	61.4 ± 10.5	57.0
<1	1,012	73.5	57.5 ± 9.5	38.2
Periodontal status				
Periodontitis	640	46.5	60.3 ± 9.8	50.9
Nonperiodontitis	737	53.5	57.1 ± 9.8	36.5

SD: standard deviation.

**Table 2:** Association of obvious reduction in salivary flow rate with its predictors determined by using logistic regression.

	Model 1	Model 2	Obvious reduction in salivary flow rate (%)	PAF (%)
	Crude OR (95% CI)	Multivariate OR^a^ (95% CI)		
Salivary flow rate (mL/min)	0.98 (0.86–1.11)		NA	NA
Age per decade^b^	1.38 (1.16–1.63)	1.25 (1.03–1.51)	NA	NA
Sex (ref. women)				
Men	1.28 (0.91–1.80)	1.11 (0.78–1.58)	12.3	9.6
Medical history of stroke	2.82 (1.25–6.40)	2.14 (0.92–4.97)	25.0	3.1
Use of antihypertensive agents	1.31 (0.90–1.91)		13.0	6.3
Smoking habits (ref. noncurrent smoker)				
Current smoker	1.08 (0.71–1.67)		11.6	1.4
Alcohol intake (ref. noncurrent drinker)				
Current drinker	0.94 (0.67–1.32)		10.6	−3.0
Tooth brushing frequency (ref. twice or more a day)				
Once a day	1.31 (0.92–1.87)		12.7	7.6
BMI (ref. <25)				
≥25	0.77 (0.51–1.15)		9.1	−6.1
Serum albumin levels [ref. normal (≥4 g/dL)]				
Low (<4 g/dL)	1.68 (1.11–2.56)	1.60 (1.04–2.46)	15.8	8.0
2 h postload plasma glucose levels [ref. nondiabetes (<200 mg/dL)]				
Diabetes (≥200 mg/dL)	1.24 (0.73–2.10)		12.8	2.1
Dental prosthetic status (ref. ≥20 remaining natural teeth without dentures)				
≥20 remaining natural teeth with dentures	1.94 (1.09–3.44)	1.48 (0.81–2.71)	17.4	10.0
<20 remaining natural teeth without dentures	2.36 (1.11–5.05)	2.06 (0.95–4.46)	20.5	
<20 remaining natural teeth with dentures	1.25 (0.74–2.13)	0.85 (0.48–1.52)	12.0	
Plaque score (ref. <1)				
≥1	1.83 (1.28–2.61)	1.53 (1.04–2.24)	15.6	15.6
Periodontal status (ref. non-periodontitis)				
Periodontitis	1.45 (1.03–2.03)	1.16 (0.81–1.66)	12.8	15.3

OR: odds ratio; CI: confidence interval; PAF: population attributable fraction; NA: not applicable; BMI: body mass index.

^a^The multivariable logistic regression model included sex and all other risk factors with a significance level of *P* < 0.05 in the univariate analysis (model 1).

^b^Age in years scaled to decades.

**Table 3:** Predictors of rate of reduction in salivary flow during the follow-up period determined by using stepwise multivariable linear regression.

	*β*	SE	Standardized *β*	*t*	*P* value
Salivary flow rate (mL/min)	0.116	0.009	0.348	12.952	<0.001
Age per decade^a^	0.058	0.011	0.132	5.100	<0.001
Sex (men = 0; women = 1)	0.116	0.024	0.132	4.853	<0.001
Plaque score (<1 = 0; ≥1 = 1)	0.065	0.026	0.066	2.526	0.012

The analysis also included use of antihypertensive agents, which was excluded in the final model.

Parameters of model: *F* = 46.858; adjusted *R* ^2^ = 0.118; *P* < 0.001.

^a^Age in years scaled to decades.
